# Lung Cancer and Cardiovascular Disease Mortality Associated with Ambient Air Pollution and Cigarette Smoke: Shape of the Exposure–Response Relationships

**DOI:** 10.1289/ehp.1103639

**Published:** 2011-07-19

**Authors:** C. Arden Pope, Richard T. Burnett, Michelle C. Turner, Aaron Cohen, Daniel Krewski, Michael Jerrett, Susan M. Gapstur, Michael J. Thun

**Affiliations:** 1 Economics Department, Brigham Young University, Provo, Utah, USA; 2 Population Studies Division, Health Canada, Ottawa, Ontario, Canada; 3 McLaughlin Center for Population Health Risk Assessment, University of Ottawa, Ottawa, Ontario, Canada; 4 Health Effects Institute, Boston, Massachusetts, USA; 5 Division of Environmental Health Science, School of Public Health, University of California, Berkeley, California, USA; 6 Epidemiology Research Program, American Cancer Society, Atlanta, Georgia, USA

**Keywords:** air pollution, cardiovascular disease, lung cancer, mortality, particulate matter, smoking

## Abstract

Background: Lung cancer and cardiovascular disease (CVD) mortality risks increase with smoking, secondhand smoke (SHS), and exposure to fine particulate matter < 2.5 μm in diameter (PM_2.5_) from ambient air pollution. Recent research indicates that the exposure–response relationship for CVD is nonlinear, with a steep increase in risk at low exposures and flattening out at higher exposures. Comparable estimates of the exposure–response relationship for lung cancer are required for disease burden estimates and related public health policy assessments.

Objectives: We compared exposure–response relationships of PM_2.5_ with lung cancer and cardiovascular mortality and considered the implications of the observed differences for efforts to estimate the disease burden of PM_2.5_.

Methods: Prospective cohort data for 1.2 million adults were collected by the American Cancer Society as part of the Cancer Prevention Study II. We estimated relative risks (RRs) for increments of cigarette smoking, adjusting for various individual risk factors. RRs were plotted against estimated daily dose of PM_2.5_ from smoking along with comparison estimates for ambient air pollution and SHS.

Results: For lung cancer mortality, excess risk rose nearly linearly, reaching maximum RRs > 40 among long-term heavy smokers. Excess risks for CVD mortality increased steeply at low exposure levels and leveled off at higher exposures, reaching RRs of approximately 2–3 for cigarette smoking.

Conclusions: The exposure–response relationship associated with PM_2.5_ is qualitatively different for lung cancer versus cardiovascular mortality. At low exposure levels, cardiovascular deaths are projected to account for most of the burden of disease, whereas at high levels of PM_2.5_, lung cancer becomes proportionately more important.

Active cigarette smoking is the major cause of lung cancer and an important established cause of cardiovascular disease (CVD) mortality (U.S. Department of Health and Human Services 2004, 2010). Risks have been shown to increase with even light or intermittent active smoking (Bjartveit and Tverdal 2005; Schane et al. 2010; U.S. Department of Health and Human Services 2010). Secondhand smoke (SHS) is also an established cause of both lung cancer and CVD (U.S. Department of Health and Human Services 2004, 2006, 2010). Since the early 1990s, growing evidence has linked long-term exposure to fine particulate matter (particulate matter with an aerodynamic diameter ≤ 2.5 μm; PM_2.5_) air pollution with increases in the risk of cardiovascular mortality (Brook et al. 2010; Dockery et al. 1993; Krewski et al. 2009; Laden et al. 2006; Miller et al. 2007; Pope et al. 1995, 2004; Pope and Dockery 2006) and, to a lesser extent, lung cancer (Chen et al. 2008; Pope et al. 2002).

In a recent analysis, we evaluated the exposure–response relationship for cardiovascular mortality in relation to PM_2.5_ from active cigarette smoking, SHS, and ambient air pollution (Pope et al. 2009). The results suggested a relatively steep exposure–response function at very low levels of exposure and a flattening out of CVD risk at high exposure levels. Previous efforts to estimate the disease burden attributable to PM_2.5_ exposure have assumed that the adverse effects of PM_2.5_ on both cardiovascular and lung cancer mortality flatten out above 50 μg/m^3^ (Cohen et al. 2004). If the lung cancer effects of PM_2.5_, however, do not flatten out at high levels of exposure similar to CVD, the estimates of the lung cancer excess disease burden from air pollution would be substantially underestimated in areas with high levels of PM_2.5_ pollution.

In the present analysis, therefore, we explicitly conducted an original evaluation of the shape of the PM_2.5_-mortality exposure–response relationship for lung cancer. For direct comparison, we also reevaluated the shape of the PM_2.5_-mortality exposure–response relationship for CVD mortality using the same cohort, follow-up period, exclusion criteria, covariates, and related statistical modeling approaches. In an integrative way, our approach evaluates exposure–response relationships using three basic sources of exposure (active smoking, passive smoking, and ambient air pollution) in relation to two major health end points (cardiovascular and lung cancer mortality), using PM_2.5_ as the common index of exposure. There are limitations and uncertainties related to using any single measure as a common index of exposure. Nevertheless, this analysis allows for direct comparisons of the exposure–response relations for lung cancer versus CVD mortality and provides the opportunity to evaluate the implications of the observed differences when estimating the disease burden from PM_2.5_.

## Methods

*Cohort data.* Estimates of adjusted relative risks (RRs) over different increments of active cigarette smoking are based on data collected by the American Cancer Society (ACS) as part of the Cancer Prevention Study II (CPS-II), an ongoing prospective cohort mortality study involving nearly 1.2 million adults. Ethics approval for the CPS-II was obtained from the Emory University School of Medicine Human Investigations Committee. More detailed descriptions of this cohort are provided elsewhere (Calle and Terrel 1993; Chao et al. 2000; Hoover et al. 2003; Krewski et al. 2009; Pope et al. 2002). Participants from throughout the United States were enrolled by > 77,000 volunteers between September 1982 and February 1983. Enrollment was restricted to persons who were ≥ 30 years of age and who were members of households with one or more members ≥ 45 years of age. At enrollment, participants completed a self-administered confidential questionnaire that captured data on a range of demographic, lifestyle, medical, and other individual characteristics (ACS 2011). Through 1988, vital status of study participants was ascertained by both personal contact and subsequently through linkage with the National Death Index (Calle and Terrel 1993). Death certificates were obtained and coded for cause of death.

Cause of death was coded according to two-digit ACS codes that were consolidations of *International Classification of Diseases, 9th Revision* (ICD-9) codes (Pope et al. 2004; World Health Organization 1977). Lung cancer deaths included deaths due to malignant neoplasms of the trachea, bronchus, and lung (ICD-9 162). As discussed elsewhere (Pope et al. 2009), cardiovascular diseases and cardiopulmonary diseases (CPDs) have substantial common comorbidity. Cross-coding and misclassification of primary causes of death are inherent in the use of death certificate data, making it unclear which ICD-9 groupings most generally or specifically indicate death due to CVD. For example, inflammation associated with pulmonary disease contributes to cardiovascular risk (van Eeden et al. 2005), and individuals with chronic obstructive pulmonary disease are likely to be coded as dying of CVD based on death certificate data (Sin et al. 2005; Speizer et al. 1989). Because of this cross-classification problem, we examined the exposure–response function for CVD deaths using three different outcome groupings, namely, *a*) ischemic heart disease (IHD) (ICD-9 410–414), *b*) CVD (ICD-9 401–459), and *c*) CPD (ICD-9 401–459 and 460–519).

Because additional information regarding cigarette smoking was not collected after enrollment, the present analysis was based on a restricted follow-up period of approximately 6 years through 31 December 1988. The unrestricted CPS-II cohort included a total of 1,184,881 participants. For this analysis, subjects were excluded if they had prevalent cancer (except nonmelanoma skin cancer) at baseline (82,329), or if they had missing or erroneous data for vital status (419), race (5,318), education (16,946), marital status (5,239), body mass index (30,291), SHS exposure (9,799), or cigarette smoking habits or history (208,835). Because this study is focused on effects of long-term exposure, we also excluded participants who started smoking after 25 years of age (30,921). The final analytic cohort included 794,784 subjects, among whom 3,194 lung cancer deaths, 11,607 IHD deaths, 19,290 cardiovascular deaths, and 22,021 cardiopulmonary deaths occurred during the approximately 6-year follow-up period.

*Statistical analysis.* Cox proportional hazards survival models (Fleming and Harrington 1991) were estimated separately for lung cancer, ischemic heart, cardiovascular, and cardiopulmonary deaths. Data from participants who died of other causes were censored at time of death. Survival time from the date of enrollment was used as the time axis. Baseline hazard functions were stratified by 1-year age categories, sex, and race (white, black, other). The models included indicator variables for smoking increments of ≤ 3, 4–7, 8–12, 13–17, 18–22, 23–27, 28–32, 33–37, 38–42, and ≥ 43 cigarettes per day for current smokers relative to never smokers. To control for smoking in previous smokers relative to never smokers, former-smoking indicator variables using the same cigarettes-per-day increments were included in the models. Additionally, the models included variables to control for education (two variables that indicate high school education or more than high school education versus less than high school education), marital status (two variables that indicate separated/divorced/widowed or single versus married), body mass (two variables representing linear and squared terms for body mass index), alcohol consumption (six variables that indicate consumption of or missed reporting of beer, wine, or other alcohol versus nondrinkers), occupational exposures [one variable indicating self-reported exposure to dust and fumes in the workplace and seven additional variables that indicate different rankings of an occupational dirtiness index versus a referent category, as has been developed and described elsewhere (Siemiatycki et al. 2003)], and diet [eight indicator variables that contrasted quintiles of dietary fat consumption and quintiles of combined consumption of vegetables, citrus, and high-fiber grains (Chao et al. 2000)].

To evaluate potential effect modification by sex, we also modeled the Cox proportional hazards estimates described above separately for males and females. To account for the impact of smoking duration on effect estimates and the shape of the exposure–response relationship, we conducted further analyses that stratified all of the cigarettes-per-day smoking increments by three levels of smoking duration. Specifically, the cigarettes-per-day smoking increment indicator variables (for current and previous smokers) were each replaced by three variables that indicated the specific cigarettes-per-day increments for three smoking durations: < 30 years, 30–39 years, and ≥ 40 years.

*Plotting exposure–response relationships.* The adjusted RRs (as estimated by the hazard ratios from the Cox proportional hazard model) associated with different increments of PM_2.5_ exposure from active cigarette smoking, SHS, and ambient air pollution were plotted against estimated average daily inhaled dose of PM_2.5_. For active smoking, the average inhaled dose was assumed to be 12 mg PM_2.5_ per cigarette. The actual amount of PM_2.5_ inhaled per cigarette is influenced more by individual inhalation and smoking patterns than by the tar level measured by machine smoking (National Cancer Institute 2001). Nevertheless, the estimated sales-weighted average of PM from cigarettes sold in the United States in the 1980s and 1990s was approximately 12–14 mg per cigarette (National Cancer Institute 2001). Because of uncertainty regarding estimates of PM_2.5_ exposure per cigarette smoked, reference exposure scaling expressed as actual increments of cigarettes smoked per day is also provided in the exposure–response plots.

Comparative estimates of excess risk of mortality from long-term exposure to ambient air pollution come from several key prospective cohort studies, including estimates from analyses using ACS CPS-II cohort participants who lived in metropolitan areas with available air pollution data (Pope et al. 1995, 2002, 2004), analyses of the Harvard Six Cities (HSC) study (Dockery et al. 1993; Laden et al. 2006), and the Women’s Health Initiative (WHI) study (Miller et al. 2007). The RRs of mortality are taken from the published estimates from these studies. The average daily dose of inhaled PM_2.5_ is estimated by multiplying the relevant average ambient PM_2.5_ concentrations by average daily inhalation rates (cubic meters per day). Actual ventilation rates depend on age, sex, body size, activity levels, and other factors. The estimated average volume of air inhaled daily by adults ranges from 13 to 23 m^3^/day (Allan et al. 2008; Brochu et al. 2006; Layton 1993; Stifelman 2007; U.S. Environmental Protection Agency 1997). Our analyses use 18 m^3^/day (Allan et al. 2008; Brochu et al. 2006; Stifelman 2007) in calculating PM_2.5_ dosage at various levels of air pollution. Based on the key prospective cohort studies cited above, the range of average ambient PM_2.5_ concentrations is approximately 5–30 μg/m^3^, resulting in estimated daily dose of PM_2.5_ from ambient air pollution potentially ranging from 0.09 to 0.54 mg. In our analyses, however, the plotted RRs represent changes in risk across contrasts in exposure that are internal to and reported by the specific studies.

Comparative estimates of excess risk of mortality from long-term exposure to SHS come from pooled estimates from the 2006 Surgeon General’s Report (SGR) (U.S. Department of Health and Human Services 2006). These pooled estimates include exposure assessments based simply on SHS exposure at home or work (for example, nonsmokers living with a spouse who smokes or nonsmokers employed in an SHS environment) and on more quantified assessment of SHS exposure including low to moderate SHS exposure (passive exposure to either 1–14 or 1–19 cigarettes per day) and moderate to high exposure (≥ 15 or ≥ 20 cigarettes per day). Additionally, risk estimates for acute myocardial infarction from the INTERHEART study (Teo et al. 2006) of 52 countries associated with 1–7 hr per week of SHS exposure or exposure from living with a spouse who smoked are included. Estimated average PM_2.5_ exposure is approximately 20 μg/m^3^ for low to moderate SHS exposure and exposure incurred over an interval of 1–7 hr per week, 50 μg/m^3^ for moderate to high exposure, 30 μg/m^3^ for living with a spouse who smokes, and 40 μg/m^3^ for working in a workplace with reported SHS exposure. These estimates are based on limited data from studies that sampled the PM_2.5_ concentrations from SHS in various settings over time (Dockery and Spengler 1985; Jenkins et al. 1996; Leaderer and Hammond 1991; Spengler 1991; Spengler et al. 1985; U.S. Department of Health and Human Services 2006). The average daily dose of inhaled PM_2.5_ from SHS is estimated by multiplying the estimated average concentrations by the inhalation rate of 18 m^3^/day.

To help illustrate the integrated exposure–response relationship, the risk and dose estimates for different increments of active smoking, SHS, and ambient PM_2.5_ were used to fit a simple power function of the form [RR = 1 + α(dose)^β^] for both lung cancer and cardiovascular mortality. This functional form was selected because it represents a simple, well-behaved, nonlinear, monotonic function that goes through the origin (RR = 1 at a dose of zero). The functions were fit using iterative nonlinear regression (PROC NLIN in SAS, release 9.2; SAS Institute Inc., Cary, NC, USA) and were plotted along with the specific point estimates.

## Results

**Figure 1:**
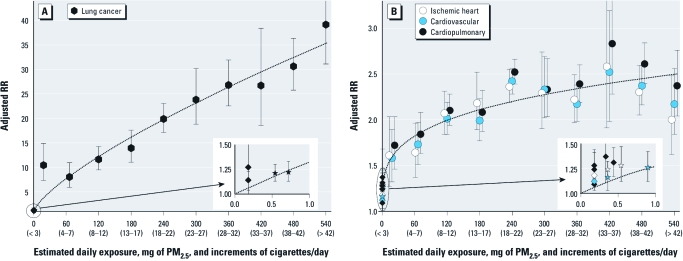
Adjusted RRs [with 95% confidence intervals (CIs)] of lung cancer mortality (*A*) and IHD, cardiovascular, and cardiopulmonary mortality (*B*) plotted over estimated daily exposure of PM_2.5_ (milligrams) and increments of cigarette smoking relative to never smokers (cigarettes/day). Diamonds represent comparative mortality risk estimates (with 95% CIs) for PM_2.5_ from air pollution from the comparative studies (Dockery et al. 1993; Laden et al. 2006; Miller et al. 2007; Pope et al 1995, 2002, 2004). Stars represent comparable pooled RR estimates (with 95% CIs) associated with SHS exposure from comparative studies (Teo et al. 2006; U.S. Department of Health and Human Services 2006). The dotted lines represent the nonlinear power function fit through the origin and the estimates (including active smoking, SHS, ambient PM_2.5_). Estimated doses from different increments of active smoking are dramatically larger than estimated doses from ambient air pollution or SHS; therefore, associations at lower exposure levels (due to ambient air pollution and SHS) are shown as insets with a magnified scale.

Table 1 presents selected summary statistics for the primary ACS CPS-II analytic cohort used to estimate the adjusted RRs for different increments of cigarette smoking. Table 2 presents adjusted RRs and estimated daily dose of PM_2.5_ for various increments of exposure from cigarette smoking, SHS, and ambient air pollution from the present analysis and the selected comparison studies. Figure 1A presents the adjusted RR estimates for lung cancer mortality plotted against estimated daily dose of PM_2.5_ from different increments of current cigarette smoking (relative to never smokers) and from different exposures from ambient air pollution and SHS. Figure 1B presents the adjusted RRs for IHD, CVD, and CPD plotted against estimated daily dose of PM_2.5_ from different increments of current cigarette smoking (relative to never smokers) and from different exposures from ambient air pollution and SHS. Because the estimated doses from different increments of active smoking are dramatically larger than estimated doses from ambient air pollution or SHS, associations at lower exposure levels (due to ambient air pollution and SHS) are shown as insets with a magnified scale. The comparative mortality risk estimates for PM_2.5_ and SHS represent changes in risk across contrasts in exposure that are internal to the specific or pooled studies.

**Table 1:** Selected summary statistics for the ACS analytic cohort.

Characteristic	Summary statistic
Total subjects in analytic cohort (*n*)	794,784
Age at enrollment [years (mean ± SD)]	56.0 ± 10.5
Body mass index (mean ± SD)	25.1 ± 4.0
Percentage of cohort	
Female	61.0
White	94.3
High school education	33.1
> High school education	53.7
Current smoker	19.9
Former smoker	26.8
Percentage of current smokers who smoked	
≤ 3 cigarettes/day	3.7
4–7 cigarettes/day	5.8
8–12 cigarettes/day	12.2
13–17 cigarettes/day	7.2
18–22 cigarettes/day	33.4
23–27 cigarettes/day	5.0
28–32 cigarettes/day	13.9
33–37 cigarettes/day	1.6
38–42 cigarettes/day	13.3
≥ 43 cigarettes/day	3.9
Percentage of current smokers with smoking duration	
< 30 years	31.2
30–39 years	38.8
≥ 40 years	30.0

**Table 2:** Adjusted RR estimates*a* for various increments of exposure from cigarette smoking (versus never smokers), SHS, and ambient air pollution from the present analysis and selected comparison studies.

Adjusted RR (95% confidence interval)							Estimated daily dose PM_2.5_ (mg)*b*
Source of risk estimate	Increments of exposure	Lung cancer	IHD	CVD	CPD		
ACS-present analysis		≤ 3 (1.5) cigarettes/day		10.44 (7.30, 14.94)		1.61 (1.27, 2.03)		1.58 (1.32, 1.89)		1.72 (1.46, 2.03)		18
ACS-present analysis		4–7 (5.5) cigarettes/day		8.03 (5.89, 10.96)		1.64 (1.37, 1.96)		1.73 (1.51, 1.97)		1.84 (1.63, 2.08)		66
ACS-present analysis		8–12 (10) cigarettes/day		11.63 (9.51, 14.24)		2.07 (1.84, 2.31)		2.01 (1.84, 2.19)		2.10 (1.94, 2.28)		120
ACS-present analysis		13–17 (15) cigarettes/day		13.93 (11.04, 17.58)		2.18 (1.89, 2.52)		1.99 (1.77, 2.23)		2.08 (1.87, 2.32)		180
ACS-present analysis		18–22 (20) cigarettes/day		19.88 (17.14, 23.06)		2.36 (2.19, 2.55)		2.42 (2.28, 2.56)		2.52 (2.39, 2.66)		240
ACS-present analysis		23–27 (25) cigarettes/day		23.82 (18.80, 30.18)		2.29 (1.91, 2.75)		2.33 (2.02, 2.69)		2.33 (2.03, 2.67)		300
ACS-present analysis		28–32 (30) cigarettes/day		26.82 (22.54, 31.91)		2.22 (1.97, 2.49)		2.17 (1.98, 2.38)		2.39 (2.19, 2.60)		360
ACS-present analysis		33–37 (35) cigarettes/day		26.72 (18.58, 38.44)		2.58 (1.91, 3.47)		2.52 (1.98, 3.19)		2.83 (2.28, 3.52)		420
ACS-present analysis		38–42 (40) cigarettes/day		30.63 (25.79, 36.38)		2.30 (2.05, 2.59)		2.37 (2.16, 2.59)		2.61 (2.40, 2.84)		480
ACS-present analysis		≥ 43 (45) cigarettes/day		39.16 (31.13, 49.26)		2.00 (1.62, 2.48)		2.17 (1.84, 2.56)		2.37 (2.04, 2.76)		540
ACS-air pol original		24.5 μg/m^3^ ambient PM_2.5_		—		—		—		1.31 (1.17, 1.46)		0.44
ACS-air pol extend		10 μg/m^3^ ambient PM_2.5_		1.14 (1.04, 1.23)		1.18 (1.14, 1.23)		1.12 (1.08, 1.15)		1.09 (1.03, 1.16)		0.18
HSC-air pol original		18.6 μg/m^3^ ambient PM_2.5_		—		—		—		1.37 (1.11, 1.68)		0.33
HSC-air pol extend		10 μg/m^3^ ambient PM_2.5_		1.21 (0.92, 1.69)		—		1.28 (1.13, 1.44)		—		0.18
WHI-air pol		10 μg/m^3^ ambient PM_2.5_		—		—		1.24 (1.09, 1.41)*c*		—		0.18
SGR-SHS		Low–moderate SHS exposure		—		—		1.16 (1.03, 1.32)		—		0.36
SGR-SHS		Moderate–high SHS exposure		—		—		1.26 (1.12, 1.42)		—		0.90
SGR-SHS		Live with smoking spouse		1.21 (1.13, 1.30)		—		—		—		0.54
SGR-SHS		Work with SHS exposure		1.22 (1.13, 1.33)		—		—		—		0.72
INTERHEART		1–7 hr/week SHS exposure		—		1.24 (1.17, 1.32)*d*		—		—		0.36
INTERHEART		Live with smoking spouse		—		1.28 (1.12, 1.47)*d*		—		—		0.54
Study name abbreviations: ACS-present analysis, the present analysis of the ACS CPS-II cohort; ACS-air pol original, the original analysis of air pollution and mortality using the ACS CPS-II cohort (Pope et al. 1995); ACS-extend, extended analyses of air pollution and mortality using the ACS CPS-II cohort (Pope et al. 2002 and 2004); HSC-air pol original, the original analysis of air pollution and mortality using the HSC cohort (Dockery et al. 1993); HSC-air pol extend, an extended analysis of air pollution and mortality using the HSC cohort (Laden et al. 2006); WHI-air pol, an analysis of air pollution and fatal and non-fatal cardiovascular events using the WHI cohort (Miller et al. 2007); SGR-SHS, SGR that provides pooled estimates for SHS (U.S. Department of Health and Human Services 2006); INTERHEART, a 52-country case–control study of tobacco use and risk of myocardial infarction (Teo et al. 2006). **a**Adjusted RR estimates are for mortality unless otherwise noted. **b**The estimated daily dose assumes an inhalation rate of 18 m^3^/day and a dose of 12 mg/cigarette. **c**First CVD event. **d**Myocardial infarction.												

The dotted lines in Figure 1A and B represent the fitted nonlinear power function. For lung cancer, the fitted function [RR = 1 + 0.3195(dose)^0.7433^] represents a monotonic, nearly linear exposure–response relationship with fairly constant marginal increases in RR with increasing exposure. As reported in Table 2 and as can be seen in Figure 1, the adjusted RR of lung cancer rises with increased exposure throughout the full observed range of exposure. For CVD (and related cause-of-death groupings) the fitted function [RR = 1 + 0.2685(dose)^0.2730^] indicates an exposure–response relationship that is substantially nonlinear, that is, much steeper at the very low levels of exposure compared with higher levels of exposure. At very low levels of exposure—within the range of exposures associated with ambient air pollution and SHS—excess mortality risks are similar for lung cancer and CVD mortality (compare the rescaled inserts in Figure 1). For lung cancer mortality, the RRs steadily increase to nearly 40 at the highest increment of cigarette smoking (> 42 cigarettes per day), whereas for CVD mortality, the RRs level off at approximately 2.0–2.5.

A similar, near-linear exposure–response function for lung cancer mortality was observed for both men and women when adjusted risk ratios were stratified by sex (results not shown). For CVD mortality, the adjusted RRs associated with smoking ≤ 3 cigarettes per day were notably lower for women than for men, but at higher levels of smoking (≥ 18–22 cigarettes/day), the adjusted RRs were somewhat higher for women than for men. However, for both men and women, the excess risk for CVD mortality tended to level off at high exposures.

Models that also accounted for different strata of smoking duration (< 30 years, 30–39 years, or ≥ 40 years) did not indicate consistent differences in the exposure–response relationship for CVD mortality, and nonlinear exposure–response relationships for all three smoking-duration strata were consistent with the exposure–response relationship for the population as a whole (data not shown). Exposure–response curves for lung cancer mortality were nearly linear for all strata of smoking duration, consistent with results for the population as a whole, but the slopes varied by smoking duration, with the exposure–response function much less steep for those with a smoking duration of < 30 years. Our ability to evaluate effects of smoking duration independent of age was limited by design, because this analytic cohort was constrained to smokers who started smoking as teenagers or young adults, and smoking duration was therefore highly correlated with age (correlation coefficient 0.83).

## Discussion

Results for CVD mortality were consistent with those from a previous analysis (Pope et al. 2009). Specifically, the exposure–response function is relatively steep at very low levels of exposure (in the range associated with exposure to SHS and ambient air pollution) and flattens out at high exposure levels (consistent with exposure from active cigarette smoking). Results from recent studies that have found surprisingly sizable reductions in acute cardiovascular events after public bans on smoking (Institute of Medicine 2010) are qualitatively consistent with our findings.

In the present analysis, we further evaluated the PM_2.5_ exposure–response relationship for lung cancer mortality and contrasted it with comparably estimated exposure–response relationships for cardiovascular mortality using the same cohort, follow-up period, statistical models, and adjustment for other risk factors. The exposure–response function for PM_2.5_ and lung cancer mortality is different than that for cardiovascular mortality in two important ways. First, the basic shapes of the exposure–response functions differ. For lung cancer mortality, excess risks rise nearly linearly throughout the full range of exposure from SHS, air pollution, and active smoking, reaching maximum RRs > 40 for heavy smokers.

Second, smoking duration appeared to have a much larger impact on lung cancer mortality than on CVD mortality. A more complete discussion of how CVD mortality risk is affected by the duration and intensity of exposure, provided elsewhere (Pope et al. 2011), suggests that most of the cardiovascular effects are associated with exposure most proximal in time and over exposure duration windows of a few years to approximately a decade. Consistent with these findings, stratifying by exposure durations of < 30 years, 30–39 years, or ≥ 40 years did not result in substantially different exposure–response relationships for CVD mortality risk, in contrast with our findings for lung cancer mortality. The importance of duration of smoking for lung cancer was established in the British Doctor’s Study (Doll and Peto 1978) and has been confirmed by more complete analyses of age, duration of smoking, and daily cigarette consumption based on the ACS CPS-II cohort (Flanders et al. 2003; Thun et al. 1997) and case–control studies of residential radon and lung cancer (Lubin et al. 2007). In the present analysis, we integrated exposure and risk information for active smoking, passive cigarette smoking, and air pollution to evaluate the shape of the exposure–response relationship. The range of duration of active smoking was constrained by design to facilitate comparisons with studies of long-term exposure to air pollution and SHS. Strong correlations between age and exposure duration, the fact that the Cox proportional hazards models strictly controlled for age (by allowing separate baseline hazards for each age, sex, race strata), and changes in baseline mortality risk with aging limit our ability to evaluate effects of exposure duration separately from effects of age. Nevertheless, these results clearly demonstrate fundamental differences in the shapes of the exposure–response functions for lung cancer mortality versus cardiovascular mortality.

The exposure–response relationship for PM_2.5_ and lung cancer mortality appears to be nearly linear. These results are consistent with previous analyses that demonstrated that lung cancer mortality risk estimates for SHS are similar to risk estimates calculated indirectly from linear extrapolations of excess risk from active smokers (Hackshaw et al. 1997). The differences in the exposure–response relationships for lung cancer versus CVD may have mechanistic implications, because the pathogenicity of PM_2.5_ for cardiovascular end points may be mediated more by the particles themselves, whereas the lung cancer hazard is thought to be mediated largely by carcinogenic compounds carried on the particles. Carcinogens found in tobacco smoke and in combustion-source air pollution are the likely agents responsible for the excess lung cancer risk. Fine particles transport many of the toxic and carcinogenic substances in smoke and may contribute to pulmonary and systemic inflammation. Chronic inflammation may promote genetic and possibly epigenetic changes that transform a normal cell through a multistep process toward malignancy. More complete reviews and discussions regarding the mechanistic pathways that particulate exposure can contribute to lung cancer and CVD are provided elsewhere (Brook et al. 2010; U.S. Department of Health and Human Services 2010).

There are at least four possible explanations for the very steep exposure–response for CVD mortality at low levels of exposure (within the ranges associated with air pollution and SHS) and the leveling off at high exposures (for active cigarette smoking): *a*) systematic scaling distortions, *b*) differential toxicities, *c*) self-selected smokers who are less susceptible to cardiovascular effects, and *d*) the occurrence of a saturation phenomenon with relatively low levels of exposure capable of activating relevant biological pathways. Use of the estimated average daily inhaled dose of PM_2.5_ as the common exposure metric to plot the exposure–response relationship as well as the potential for systematic measurement error in PM_2.5_ exposure estimates could result in scaling distortions. For example, information on cigarette smoking and residence history was not collected after enrollment in the ACS cohort. Changes in smoking habits, including quitting smoking, may result in underestimation of effects, although this bias would likely be minimal over the short 6-year follow-up. Furthermore, changes in cigarette design and compensatory smoking behavior may have changed the inhaled dose of PM_2.5_ per cigarette. Changes in assumptions used to calculate doses (such as ventilation rates, yields of PM_2.5_ per cigarette, and estimates of average concentrations from SHS) would alter the exposure estimates and dose scaling presented in Figure 1. Although it is possible that systematic scaling issues would fundamentally influence the shape of the exposure–response relationship, we used the same exposure scaling for lung cancer and cardiovascular mortality, and the fact that the lung cancer exposure–response was nearly linear suggests that the exposure scaling may be reasonable. For lung cancer, the primary anomaly was the relatively high adjusted RR (approximately 10) estimated for very light smokers (≤ 3 cigarettes/day).

The shape of the CVD mortality exposure–response relationship might also reflect variation in biological mechanisms, characteristics of exposure, and the toxicity of PM_2.5_ depending on the composition of fine particles from different sources—from active smoking, SHS, or air pollution. Components or combinations of components of fine particulates from cigarette smoke and other combustion sources, as well as the mechanisms responsible for the observed adverse cardiovascular health effects, have yet to be fully elucidated. However, the differential toxicity explanation is not fully consistent with the fact that in the range of exposures associated with active smoking alone the exposure–response curve is remarkably flat and, if extrapolated back to zero, would not go through the origin. Assuming there is no excess risk at zero exposure (i.e., that the dose–response curve should go through the origin in the absence of exposure), a monotonic exposure–response function based only on the evidence of excess risk due to active smoking would require a nonlinear function that is steeper at low exposures and flattens out at high exposures. This explanation could be tested further in future analyses that include estimates of cardiovascular mortality associated with exposures to high concentrations of fine particles from diverse combustion and noncombustion sources. Studies of occupational groups, such as underground miners exposed to diesel exhaust and women exposed to high levels of PM from household fuel combustion, both coal and biomass, could potentially provide such estimates.

The third potential explanation for the leveling off of the exposure–response relationship for CVD mortality in the range of exposures from active smoking is that individuals who are less susceptible to adverse cardiopulmonary effects of smoking may be more likely to smoke and continue smoking than those who are more susceptible. Exposure to ambient air pollution (and SHS, to a lesser degree) is largely involuntary and therefore is not likely to be biased by self-selection. This explanation implies that in the absence of self-selection, the relative effects of high exposures from active smoking would be larger than estimated in this analysis. However, this explanation also assumes that self-selection would not be relevant to lung cancer susceptibility. In addition, similar to the discussion of differential toxicity above, this explanation is not fully consistent with the fact that in the range of exposure due to active smoking alone, the exposure–response function is remarkably flat.

The fourth potential explanation regarding the steep exposure–response for CVD mortality at low levels of exposure and the leveling off at high exposures is a saturation phenomenon whereby relatively low levels of exposure are capable of activating relevant biological pathways. There is substantial and growing evidence that long-term exposures to PM_2.5_ from cigarette smoke, ambient air pollution, or both affect multiple physiologic pathways. Even low levels of exposure from SHS and ambient air pollution have been associated with pulmonary and systemic oxidative stress, inflammatory vascular dysfunction, increased platelet activation and blood viscosity, atherosclerosis, IHD, and altered cardiac autonomic function (Ambrose and Barua 2004; Brook et al. 2010; Pope et al. 2004; U.S. Department of Health and Human Services 2006, 2010). An evaluation of the pathophysiology of cigarette smoke and CVD suggests that underlying biochemical and cellular processes may become saturated with small doses of toxic components (Ambrose and Barua 2004). A recent study, for example, reported small airway epithelium responses (transcriptome modifications) to even the lowest levels of cigarette smoke exposures (Strulovici-Barel et al. 2010). There is evidence that relevant biological pathways for CVD may be activated at low levels of exposure and that increasing exposure further increases risk, but at a decreasing marginal rate.

The empirical findings of this analysis have important public health implications (Smith and Peel 2010). Better quantification of the exposure–response gradients across environmentally relevant ranges of exposure helps inform public health policy decisions. Estimates of the burden of disease attributable to air pollution are sensitive to assumptions regarding the shape of the exposure–response relationship, but most of the studies of the effects on cardiovascular mortality of long-term exposure to PM_2.5_ have been conducted in areas where annual average concentrations range between approximately 5 and 35 μg/m^3^. Average concentrations of particulate air pollution in major population centers of China, India, and other developing countries are often much higher, exceeding 100 μg/m^3^ according to recent estimates (Sivertsen 2006; van Donkelaar et al. 2010). For CVD, inappropriate extrapolations of linear exposure–response functions may result in substantial overestimates in areas with very high exposures and, in some cases, potential underestimates in areas with relatively low exposures. These results confirm our previous finding (Pope et al. 2009) that, for CVD, the exposure–response relationship is nonlinear, with a steep increase in risk at low exposures and flattening out at higher exposures. These results, however, indicate a substantively different exposure–response relationship for lung cancer mortality and suggest that it may be reasonable to assume a near linear exposure–response function when estimating burdens of disease across a wide range of exposures.
